# Spin-dependent Transport Properties of CrO_2_ Micro Rod

**DOI:** 10.1007/s40820-014-0010-8

**Published:** 2014-09-19

**Authors:** Zhen Wang, Li Xi, Yikai Yang, Yue Li, Xuemeng Han, Yalu Zuo, Jianbo Wang

**Affiliations:** Key Laboratory for Magnetism and Magnetic Materials of Ministry of Education, Lanzhou University, Lanzhou, 730000 People’s Republic of China

**Keywords:** CrO_2_ rod, Low-field magnetoresistance, Tunneling barrier, Bias voltage dependence

## Abstract

The CrO_2_ micro rod powder was synthesized by decomposing the CrO_3_ flakes at a specific temperature to yield precursor and annealing such a precursor in a sealed glass tube. The magneto-transport properties have been measured by a direct current four-probe method using a Cu/CrO_2_ rods/colloidal silver liquid electrode sandwich device. The largest magnetoresistance (MR) around ~72 % was observed at 77 K with applied current of 0.05 μA. The non-linear *I*–*V* curve indicates a tunneling type transport properties and the tunneling barrier height is around 2.2 ± 0.04 eV at 77 K, which is obtained with fitting the non-linear *I*–*V* curves using Simmons’ equation. A mixing of Cr oxides on the surface of CrO_2_ rod observed by X-ray photoemission spectroscopy provides a tunneling barrier rather than a single phase of Cr_2_O_3_ insulating barrier. The MR shows strong bias voltage dependence and is ascribed to the two-step tunneling process.

## Introduction

Oxide half metallic materials in which electrons express only one spin orientation at the Fermi level, such as CrO_2_, La_1−x_A_x_MnO_3_ (A = Sr, Ca, etc.), and Fe_3_O_4_, show the advantages of high spin polarization, environmental stability, and efficient spin injection which are expected to be superior to pure metals as a spin injection source [1]. Traditionally, CrO_2_ with relatively high Curie temperature of 398 K and theoretical saturation magnetization around 2 μB/f.u. was used as information storage materials for magnetic recording tapes. Nowadays, CrO_2_ attracted wide interesting for its half metallic properties, such as the double exchange coupling interaction due to the self doping effect [2], the spin-dependent magneto-transport properties in cold-press CrO_2_ powder [3, 4], and polycrystalline CrO_2_ thin films with large magnetoresistance (MR) effect [5], since it has been experimentally confirmed to exhibit almost 100 % spin polarization near the Fermi level at least in low temperatures [6, 7]. Moreover, Chromium dioxide is a meta-stable oxide of Cr ion and it can be easily further reduced to Cr_2_O_3_ in the surface of CrO_2_ [4, 5, 8]. The formed surface Cr_2_O_3_ layer was treated as a tunneling barrier between CrO_2_ particles in the spin-dependent transport measurements due to its antiferromagnetic insulating properties by the super-exchange coupling among Cr ions. Recently, Bajpai et al. also have reported related magnetoelectric effect due to the antiferromagnetic and magnetoelectric Cr_2_O_3_ layer formation in the surface of CrO_2_ [9, 10] and Zhang et al. reported the magnetic properties of epitaxial CrO_2_ thin films grown on TiO_2_ (001) substrates [11].

In this work, the rarely reported bias voltage-dependent spin transport properties of CrO_2_ micro rods are investigated. The naturally formed Cr oxidation layer on the surface of CrO_2_ micron rods was investigated by X-ray photoemission spectroscopy (XPS). A Cu/CrO_2_ rods/silver paint electrode sandwich device, in which the particles are naturally coalesced by the van der Waals force, was utilized for magneto-transport measurement [12]. The barrier thickness and barrier height based on the formation of other Cr oxidation layer in the surface of CrO_2_ were investigated. Furthermore, a two-step tunneling mechanism was used to understand the strong bias dependence of MR effect.

## Experimental

The CrO_2_ rod powder was synthesized by decomposing the CrO_3_ flakes at a specific temperature to yield precursor. Such a precursor is sealed in a quartz-glass tube with hydrogen brazing and annealed at 395 °C for 3 h [13, 14]. The crystal structure, morphology, and microstructure of the sample were characterized by a Rigaku D/Max-2400 powder x-ray diffraction (XRD) using Cu Kα radiation, a Joel 6610 scanning electron microscopy (SEM) and a FEI G2 F30S transmission electron microscope (TEM) with an accelerating voltage of 300 kV, respectively. XPS was performed using a Kratos Axis Ultra DLD photoelectron spectrometer with a monochromatic aluminum Kα source at 300 W (20 mA × 15 kV) and pass energy of 20 eV for high-resolution scans. In order to investigate the surface layer properties of CrO_2_ sample, an ion beam etching process with the source of Argon and the rotation of sample for steps of 30 s was used after obtaining a set of XPS spectrum. The static magnetic properties were characterized by a Lakeshore 7304 vibrating sample magnetometer (VSM) and a superconducting quantum interference device (SQUID).

The transport properties of CrO_2_ rod powder were investigated through a Cu/CrO_2_ rods/silver paint electrodes sandwich device which was also used in our previous work [12]. It should be mentioned that the device was vacuumed by a mechanical pump after putting CrO_2_ rods into the hole and spreading the silver paint to eliminate the adsorbed moisture and chemical solvents. A direct current four-terminal method with various bias voltages was used to conduct the transport measurement in a vacuum chamber with a Dewar filled in liquid nitrogen. A Keithley 220 current source was used to apply a constant current between the top and bottom electrode. A Keithley 2000 multimeter was used to detect the voltage between another two top and bottom electrodes.

## Results and Discussion

**Fig. 1:**
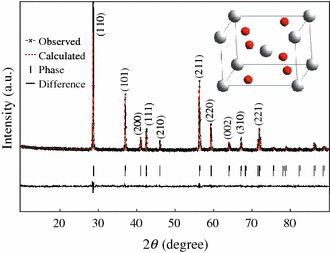
Observed (*x mark*) and calculated (*dash line*) X-ray intensity profile for CrO_2_ sample. The *inset*is a typical rutile structure calculated from the observed XRD data, where *big white* and *small red ball*, respectively represent Cr and O atoms. (Color figure online)

X-ray diffraction was used to determine the crystal structure of the obtained samples. The experimental and calculated XRD patterns, their difference and the expected peak positions of CrO_2_ rod powder based on Rietveld method refinement with P42/mnm space group, are shown in Fig. 1. It can be seen that there are no other chromium oxides phases appeared and all of the diffraction peaks correspond to the PDF card #841819 of CrO_2_. A typical rutile structure calculated from the XRD observed data via J2006 and Diamond 3.2 is obtained in the inset of Fig. 1. The XRD pattern can be well indexed using tetragonal P42/mnm space group with lattice parameters *a* = *b* = 4.423 Å, *c* = 2.918 Å. The lattice parameters are quite close to the reported values [7, 15]. It indicates that a quite pure phase of CrO_2_ rod powder was obtained in this work.

**Fig. 2:**
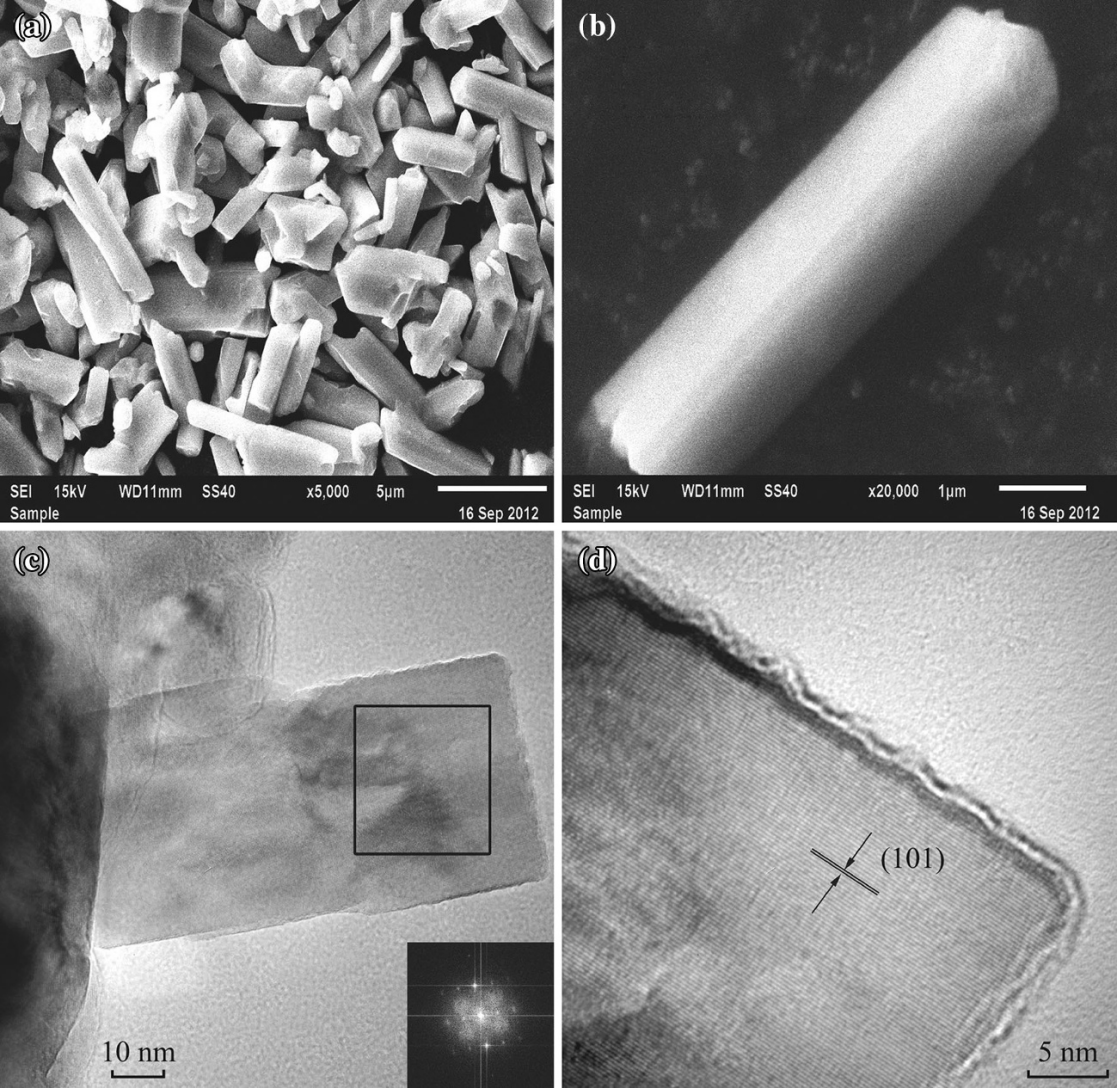
SEM images of the CrO_2_ sample **a** and **b**, TEM image **c** and high-resolution TEM image of CrO_2_ rod **d**. The *inset* of **c** is Fourier transformation of the block area

From the SEM images in Fig. 2a, b, one can see the appearance of CrO_2_ powder sample of a typical rod shape and the aspect ratio of the rod is 5:1 or less, which is different from those of 8:1 or 9:1 in the previous reports [3, 16]. The typical rod size is about 5 μm long and 1.2 μm wide. TEM images of small CrO_2_ rods are exhibited in Fig. 2c, d. The inset of Fig. 2c gives the Fourier transformation of the block area in Fig. 2c, whose pattern is consistent with the structural feature of tetragonal single crystal. Figure 2d shows a high-resolution TEM image of CrO_2_ rod, from which one can see the one-dimensional illustration of atomic array of CrO_2_ rod. The distance between the two nearby lattice arrays is 2.449 Å, which is quite close to the interplanar spacing of the (101) plane of CrO_2_. TEM observation is consistent with the result of XRD investigation.

**Fig. 3:**
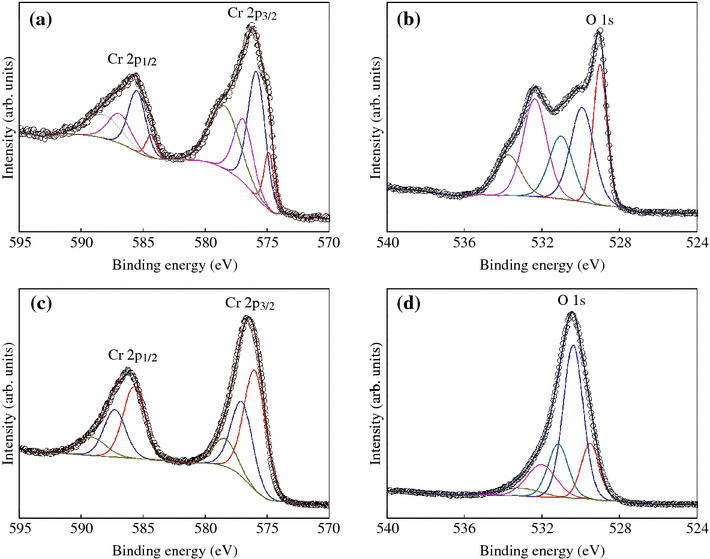
Cr 2p and O 1s core level XPS spectra for CrO_2_ sample with Ar ion beam etching for 0 (**a, b**) and 30 s (**c, d**)

The surface chemical state of CrO_2_ rods was investigated by XPS through Ar ion beam etching with sample rotating speed around 15 rad/s to uniformly remove the surface layer of powder samples. Figure 3 shows the Cr 2p and O 1 s core level XPS spectra of CrO_2_ sample at room temperature as a function of etching time. For the sample without etching, the deconvolution of Cr 2p3/2 (2p1/2) and O 1s is shown in Fig. 3a, b, respectively. The peak at 574.9 (584.4) eV corresponds to the metal Cr, which may be concluded to the defects in the surface [17]. The peak at 575.9 (585.5) eV is related to CrO_2_ [18, 19]. The peaks at 576.9 (586.9) eV and 578.5 (588.2) eV correspond to Cr_2_O_3_ and CrO_3_, respectively [17]. The peak of O 1s can be fitted by muti-peaks, and the deconvolution shows different chemical states of O in the surface of CrO_2_ rods. O 1s with binding energy of 529.0 eV is derived from CrO_2_. The peaks at 529.9 and 531.0 eV correspond to the oxides from Cr_2_O_3_ and CrO_3_, respectively [18, 19]. The peaks at 532.4 and 533.7 eV belong to the absorption of oxygen. After 30 s etching, the shoulders in the main peak of Cr 2p_3/2_ (2p_1/2_) and O 1s are all disappeared in Fig. 3c, d, indicating the surface layer of CrO_2_ rod is effectively removed by etching. From the deconvolution of Cr 2p_3/2_ (2p_1/2_), the peak at 576.0 (585.7) eV comes from CrO_2_ [18, 19]. The peaks at 577.0 (587.3) and 578.4 (589.2) eV are corresponded to the oxides from Cr_2_O_3_ and CrO_3_ [17]. The peak of O 1s at 529.5 eV is come from CrO_2_. The peaks at 530.4 and 531.2 eV are corresponded to the oxides from Cr_2_O_3_ and CrO_3_, respectively [18, 19]. The peaks at 532 and 533.1 eV are come from adsorbates and their intensities become lower after etching. Moreover, the intensities of the peaks from Cr_2_O_3_ and CrO_3_ become weak after etching. The deconvolution of Cr 2p_3/2_ (2p_1/2_) and O 1s suggests that the surface of the CrO_2_ rod sample contains a mixing of Cr oxides rather than the reported formation of single phase of Cr_2_O_3_ in the previous works [4, 5, 8]. Thus, a mixing of Cr oxides exists in the surface of CrO_2_, and this may play a tunneling barrier between the CrO_2_ rods.

**Fig. 4:**
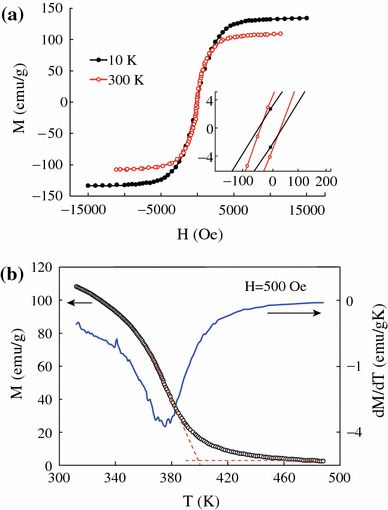
**a** Hysteresis loops of CrO_2_ powder at 300 and 10 K. **b** Temperature-dependent magnetization in a field of 500 Oe

Figure 4 shows the hysteresis loops of CrO_2_ powder at 300 and 10 K. It can be seen that the saturation magnetization increases from 108 to 133 emu/g with temperature decreasing from 300 to 10 K. The saturation magnetization at 10 K is similar to the theoretical value of 2.0 μB/f.u. for CrO_2_, indicating the pure phase of the sample and the quite thin layer of other Cr oxidation in the CrO_2_ rods surface. The hysteresis loops show small coercive force of 35 Oe at 300 K and 51 Oe at 10 K which is similar to that of epitaxial thin films of CrO_2_ [20]. Figure 4b shows the temperature dependence of the magnetization in an applied field of 500 Oe. The Curie temperature (*T*_*C*_) of the CrO_2_ is about 396 K in the previous reports [16]. Two different methods were used to determine *T*_*C*_: (i) a linear extrapolation of the *M*(*T*) to zero magnetization and (ii) the first derivative of the *M*(*T*) curves as shown in Fig. 4b. *T*_*C*_ is around 399 and 376 K from method (i) and (ii), respectively. The values of *T*_*C*_ obtained from the linear extrapolation are about 23 K higher than those calculated from the derivative method. This difference of *T*_*C*_ results from that the extrapolated values correlates to the contribution from the strongest magnetic interactions, while the derivative approach is related to the average intensity of magnetic interactions [21, 22].

**Fig. 5:**
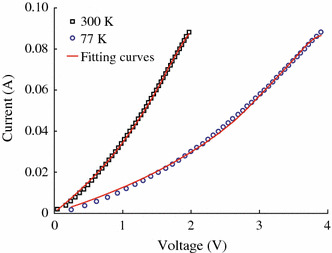
*I*-*V* curves of CrO_2_ at room temperature and 77 K

Regarding to the transport properties, the conductivity in this kind of powder heterogeneous system is determined by the chains of granules with a maximum probability of tunneling between neighboring grains. The number of such chains decreases with decreasing temperature and voltage. Heterogeneity will grow, and in the limit of very low temperatures and applied voltages, the resistance of the entire system can even be determined by a single conductive chain. However, considering CrO_2_ rod-shaped powder having large size around several microns in this work, the percolation effect can be neglected. Figure 5 shows the typical *I*–*V* curves of CrO_2_ rod powder at 300 and 77 K without magnetic field. The non-linear *I*–*V* curve indicates that there is indeed a barrier among particles. It can be seen that the nonlinearity of *I*–*V* curve becomes stronger at 77 K. According to the tunneling theory and Simmons’ equation [23],

1 $$\text{I}=\text{A}\left(\frac{\text{e}}{2\pi {\text{hd}}^{2}}\right)\left\{\left(\phi -\frac{\text{eV}}{2}\right)\exp\left[-\frac{4\pi \text{d}}{\text{h}}{\left(2\text{m}\right)}^{\frac{1}{2}}{\left(\phi -\frac{\text{eV}}{2}\right)}^{\frac{1}{2}}\right]-\left(\phi +\frac{\text{eV}}{2}\right)\exp\left[-\frac{4\pi \text{d}}{\text{h}}{\left(2\text{m}\right)}^{\frac{1}{2}}\left(\phi +{\frac{\text{eV}}{2}}^{\frac{1}{2}}\right)\right]\right\}$$

where e is the charge of the electron, m is the mess of the electron, and h is the Plank’s constant. One can fit *I*–*V* curve to extract the junction area A, the mean barrier height*ϕ*, and the barrier thickness d for a magnetic tunnel junction [23, 24]. In this work, the adjacent of two CrO_2_ rods can be considered as a tunnel junction since a mixing of Cr oxides layer formation in the surface of CrO_2_ rod observed by XPS. The electron transport is through the network of CrO_2_ junctions. We roughly assume that the number of series junctions is equal to that of parallel junctions and all tunnel junctions are the same, then the *I*–*V* character of the network junctions is equal to that of one tunnel junction. Then we can fit *I*–*V* curves of CrO_2_ rod at 300 and 77 K using Simmons equation with *A*, *ϕ* and d as the fitting parameters. The fitting results are shown in Table 1. For a real tunnel junction, the junction area and barrier thickness should be temperature independent. While in this work, the effective junction area and the thickness of the effective barrier become small with temperature increasing, which may be ascribed to assumption of the contraction of CrO_2_ rod at low temperature. This assumption can explain the increased barrier height with the decreasing of the temperature. However, there is a lack of experimental data on the temperature dependence of the thermal expansion coefficients of CrO_2_.

**Table 1:** The fitting parameters based on Simmons’ model at 300 and 77 K

Temperature (K)	*A*(μm^2^)	*d*(nm)	*ϕ*(eV)
300	0.29 ± 0.006	5.19 ± 0.05	1.16 ± 0.02
77	0.08 ± 0.002	4.13 ± 0.04	2.20 ± 0.02

**Fig. 6:**
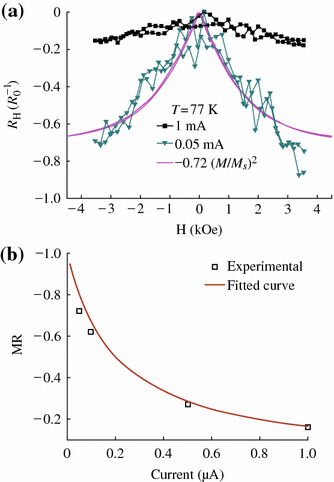
**a** Magnetoresistance curve with different applied currents. **b** The applied current dependence of MR at 77 K

Magneto-transport properties of CrO_2_ particles at 77 K and various currents are shown in Fig. 6. We define that the MR is equal to *R*(*H*)/*R*(*H*_*0*_)-1, in which *R*(*H*) and *R*(*H*_*0*_) standing for the resistance at field H and zero field, respectively. The low-field MR (LFMR) always occurs at low magnetic field (<5,000 Oe). At room temperature, the LFMR is not observed. While, at 77 K, the LFMR of CrO_2_ rod is ~16 and ~72 % with applied different currents of 1.0 and 0.05 μA at 3,500 Oe, respectively. The fluctuation of the MR data point in Fig. 6a may be caused by the displacement of the CrO_2_ rod, because the magnetic field force is larger than the van der Waals force since the rod sample with large size and strong magnetization. It can be seen that the LFMR ratio decreases as the applied current increasing, which is a typical behavior for magnetic tunnel junctions. This strong bias dependence of LFMR also proves that the MR is an inherent property of the tunneling barrier.

The process of resistance with the change of the applied magnetic field corresponds to the hysteresis loop, and the maxima MR always happen at *H*_*c*_, as a result,

2 $$\left[\rho \left(H\right)-\rho \left(H_{c}\right)\right]/\rho \left(H_{c}\right)\approx \alpha {\left(M/M_{s}\right)}^{2}$$

*α* is relative to the ultimate size of the MR. Its magnitude depends on spin-dependent scattering, *M* is the global magnetization, and *M*_*s*_ is the saturation magnetization [25]. Figure 6a also shows the square of the normalized magnetization ~0.72(*M*/*M*_*s*_)^2^ (solid line). This curve almost follows the MR curve, which suggests that the LFMR is induced by spin-dependent transport. Thus, LFMR must come from the spin-dependent tunneling between the CrO_2_ rods through other Cr oxides. According to the Jullière’s model and considering the randomly oriented magnetic moment at the coercive field for a system of non-interacting nanoparticles assemblies, Maekawa et al. derived a theoretical expression of the TMR = −*P*^2^, where *P* is the spin polarization of the magnetic material [26]. Then, we can estimate the spin polarization of the CrO_2_ rod roughly to *P*~84.9 % at 77 K. The two-step tunneling model based on a second tunneling via defect states in the barrier, in addition to the spin-dependent direct tunneling, is proposed to account for the MR-*V* dependence. The model assumes that the defect states in the barrier are uniformly distributed, both spatially and energetically, and the energy distribution of the available defect states is governed by a Fermi–Dirac function [27]. Similarly, for CrO_2_ rod powder, the tunneling barrier is mainly the space between the rods and the other chromium oxides on the surface, and this barrier can be more easily affected by the temperature and voltage compared defect states in the barrier of tunneling junctions. Thus, the bias dependent of MR can be fitted as shown in Fig. 6b. The consistence of the experimental data and the fitting curve indicates that the current in CrO_2_ rod is mainly carried by the spin-dependent direct tunneling at low bias voltage in contrast with the spin-independent two-step tunneling through barrier defects at high bias voltage. Spin-dependent magneto-transport properties of CrO_2_ with different structures have been studied by different groups as shown in Table 2. Comparing to those results, one can see that the CrO_2_ rods have shown a higher MR value with a lower applied field in this work.

**Table 2:** Magnetoresistance of CrO_2_

	MR (%)	T(K)	Applied field (Oe)	Ref.
CrO_2_ polycrystalline film	27	5	50,000	[5]
Cold pressed CrO_2_	50	5	50,000	[3]
Aligned CrO_2_ powder	41	5	1,000	[4]
CrO_2_ rod	72	77	3,500	This work

## Conclusions

In summary, we have fabricated single-phased CrO_2_ rod powder. The largest MR around ~72 % was observed using a Cu/CrO_2_ powder/silver paint electrode sandwich device at 77 K. The tunneling barrier height is around 2.2 ± 0.04 eV at 77 K, which is obtained by fitting the non-linear *I*–*V* curves using Simmons’ equation. A mixing of Cr oxides on the surface of CrO_2_ observed by XPS provides a tunneling barrier in contrast with a single phase of Cr_2_O_3_ insulating barrier as reported in the previous report. The MR shows strong bias voltage dependence and is ascribed by the two-step tunneling process. The quite thin mixing Cr oxides layer on the surface of the CrO_2_ may have defects, in which the two-step tunneling process happening at high bias voltage and resulting in the strong decay of magnetoresistance with the increase of bias voltage.

## References

[1] Soulen RJ, Byers JM, Osofsky MS, Nadgorny B, Ambrose T, Cheng SF, Broussard PR, Tanaka CT, Nowak J, Moodera JS, Barry A, Coey JMD (1998). Measuring the spin polarization of a metal with a superconducting point contact. Science.

[2] Shim JH, Lee S, Dho J, Kim DH (2007). Coexistence of two different Cr ions by self-doping in half-metallic CrO_2_ nanorods. Phys. Rev. Lett..

[3] Coey JMD, Berkowitz AE, Balcells L, Putris FF (1998). Magneto resistance of chromium dioxide powder compacts. Phys. Rev. Lett..

[4] Dai J, Tang J (2001). Junction-like magneto resistance of intergranular tunneling in field-aligned chromium dioxide powders. Phys. Rev. B.

[5] Hwang HY, Cheong SW (1997). Enhanced intergrain tunneling magnetoresistance in half-metallic CrO_2_ films. Science.

[6] Schwarz K (1986). CrO_2_ predicted as a half-metallic ferromagnet. J. Phys. F.

[7] Anguelouch A, Gupta A, Xiao G, Abraham DW, Ji Y, Ingvarsson S, Chien CL (2001). Near-complete spin polarization in atomically-smooth chromium-dioxide epitaxial films prepared using a CVD liquid precursor. Phys. Rev. B.

[8] Dai J, Tang J, Xu H, Spinu L, Wang W, Wang K, Kumbhar A, Li M, Diebold U (2000). Characterization of the natural barriers of intergranular tunnel junctions: Cr_2_O_3_ surface layers on CrO_2_ nanoparticles. Appl. Phys. Lett..

[9] Bajpai A, Borisov P, Gorantla S, Klingeler R, Thomas J, Gemming T, Kleemann W, Büchner B (2010). Interface-driven magnetoelectric effects in granular CrO_2_. Europhys. Lett..

[10] A. Bajpai, R. Klingeler, N. Wizent, A.K. Niga, S.W. Cheong, B. Büchner, Unusual field dependence of remanent magnetization in granular CrO_2_: the possible relevance of piezomagnetism. J. Phys.: Cond. Matt. **22**(9), 096005 (2010). doi:10.1088/0953-8984/22/9/096005 10.1088/0953-8984/22/9/096005 21389432

[11] Zhang X, Zhong X, Visscher PB, LeClair PR, Gupta A (2013). Structural and magnetic properties of epitaxial CrO_2_ thin films grown on TiO_2_ (001) substrates. Appl. Phys. Lett..

[12] Xi L, Du JH, Ma JH, Wang Z, Zuo YL, Xue DS (2013). Spin-dependent transport properties of oleic acid molecule self-assembled La 0.7Sr_0.3_MnO_3_ nanoparticles. J. Alloy. Compd..

[13] Bajpai A, Nigam AK (2005). Synthesis of high-purity samples of CrO_2_ by a simple route. Appl. Phys. Lett..

[14] Xi L, Yang YK (2011). Ultrawide band microwave absorption properties of ultrasound processed CrO_2_–paraffin wax composites. Jpn. J. Appl. Phys..

[15] Korotin MA, Anisimov VI, Khomskii DI, Sawatzky GA (1998). CrO_2_: a self-doped double exchange ferromagnet. Phys. Rev. Lett..

[16] G.P. Singh, S. Ram, J. Eckert, H.J. Fecht, Synthesis and morphological stability in CrO_2_ single crystals of a half-metallic ferromagnetic compound. J. Phys.: Conf. Ser. **144**(1), 012110 (2009). doi:10.1088/1742-6596/144/1/012110

[17] Halada PG, Clayton RC (1991). Photoreduction of hexavalent chromium during X-Ray photoelectron spectroscopy analysis of electrochemical and thermal films. J. Electrochem. Soc..

[18] Bullen HA, Garrett SJ (2002). Epitaxial growth of CrO_2_ thin films on TiO_2_ (110) surfaces. Chem. Mater..

[19] Heinig NF, Jalili H, Leung KT (2007). Fabrication of epitaxial CrO_2_ nanostructures directly on MgO (100) by pulsed laser deposition. Appl. Phys. Lett..

[20] Li XW, Gupta A, Giang X (1999). Influence of strain on the magnetic properties of epitaxial (100) chromium dioxide (CrO_2_) films. Appl. Phys. Lett..

[21] Navarro J, Nogues J, Muñoz JS, Fontcuberta J (2003). Antisites and electron-doping effects on the magnetic transition of Sr_2_FeMoO_6_ double perovskite. Phys. Rev. B.

[22] Chen L, Yuan CL, Xue JM, Wang J (2006). Enhancement of magnetization and curie temperature in Sr_2_FeMoO_6_ by Ni doping. J. Am. Ceram. Soc..

[23] Simmons JG (1963). Generalized formula for the electric tunnel effect between similar electrodes separated by a thin insulating film. J. Appl. Phys..

[24] Tan RP, Carrey JL, Respaud M (2008). Voltage and temperature dependence of high-field magnetoresistance in arrays of magnetic nanoparticles. J. Appl. Phys..

[25] Xiao JQ, Jiang JS, Chien CL (1992). Giant magnetoresistance in nonmultilayer magnetic systems. Phys. Rev. Lett..

[26] Inoue J, Maekawa S (1996). Theory of tunneling magnetoresistance in granular magnetic films. Phys. Rev. B.

[27] Zhang J, White RM (1998). Voltage dependence of magnetoresistance in spin dependent tunneling junctions. J. Appl. Phys..

